# Optimum Integration Procedure for Connectionist and Dynamic Field Equations

**DOI:** 10.3389/fnbot.2021.670895

**Published:** 2021-05-28

**Authors:** Andrés Rieznik, Rocco Di Tella, Lara Schvartzman, Andrés Babino

**Affiliations:** ^1^Consejo Nacional de Investigaciones Científicas y Técnicas (CONICET), Buenos Aires, Argentina; ^2^INCYT, CONICET-INECO, Buenos Aires, Argentina; ^3^El Gato y La Caja, Buenos Aires, Argentina; ^4^Facultad de Ciencias Exactas y Naturales, Universidad de Buenos Aires (UBA), Buenos Aires, Argentina; ^5^Integrative Neuroscience Lab, The Rockefeller University, New York, NY, United States

**Keywords:** cognitive neuroscience, connectionism and neural nets, neurorobotic, cognitive models, numerical optimisation techniques

## Abstract

Connectionist and dynamic field models consist of a set of coupled first-order differential equations describing the evolution in time of different units. We compare three numerical methods for the integration of these equations: the Euler method, and two methods we have developed and present here: a modified version of the fourth-order Runge Kutta method, and one semi-analytical method. We apply them to solve a well-known nonlinear connectionist model of retrieval in single-digit multiplication, and show that, in many regimes, the semi-analytical and modified Runge Kutta methods outperform the Euler method, in some regimes by more than three orders of magnitude. Given the outstanding difference in execution time of the methods, and that the EM is widely used, we conclude that the researchers in the field can greatly benefit from our analysis and developed methods.

## Introduction

Recent research indicates that connectionist and dynamic field theory (DFT) models might push developments in various branches of robotics (Schürmann et al., 2019; Tekülve et al., 2019; Schürmann and Beckerle, 2020; Torricelli et al., 2020) and specifically in embodied artificial cognitive systems (Lomp et al., 2016). Optimum integration procedures for these models may greatly contribute to the development of proper architectures, by accelerating simulation times, or equivalently, by reducing numerical errors.

For instance, when DFT architectures are used in artificial cognitive systems that are tied to real sensory data and drive autonomous robots, the relation between the physical time, when the computer provides a new value for the dynamical variables, and the simulated time is important. Ideally, computation time is not a concern because the computer is fast enough to provide updates within the time interval that is an adequate time step for the dynamics with the desired time scales. But, if the computer systematically takes longer to provide an update of the dynamic variables than the simulated time, then the dynamics cannot be realized on the artificial cognitive system. Open software frameworks as *cedar* typically address these cases by decreasing the simulation time at the next update, by increasing the integration time step, to bring the simulation time back in line with physical time. But given that DFT architectures can become large and complex, potentially including dozens of fields of different dimensionality, this procedure may lead to prohibitively large errors (Lomp et al., 2016).

Another example comes from the field of mathematical cognition, where a well-known non-linear connectionist model is used to simulate response times and error patterns in single-digit multiplication (Verguts and Fias, 2005). This model explains why, for instance, universally the most common mistake when retrieving 7 × 8 is to answer 54 instead of 56. In order to verify that this model produces observed pattern of human responses, thousands of simulations with different input values and noise levels are required. Also, if this model, as suggested in recent articles (Campbell et al., 2015; Bellon et al., 2016), should be extended or modified to explain phenomena such as retrieval-induced interference between multiplications and additions (Campbell et al., 2015) or inhibition related to individual differences (Bellon et al., 2016), it is clear that the exploration of different tentative architectures require optimum integration procedures to be practical, taking reasonable simulation times.

To the best of our knowledge, in order to simulate connectionist and dynamic field models the Euler Method (EM) is widely used. It was used, for instance, in Thelen et al. (2001) (as explicitly stated in page 21, second paragraph), in Verguts and Fias (2005) (according to a personal communication), in Lomp et al. (2016), and in Tekülve et al. (2019). In Lomp et al. (2016), the authors explicitly stated that they use the EM and did not use higher-order numerical methods because they require very many function evaluations per time step, which defeats their computational advantage when each evaluation is computationally costly.

Here we introduce two alternative numerical methods (a modified version of the fourth-order Runge Kutta method, and one semi-analytical method) and apply them to solve a well-known non-linear connectionist model of retrieval in single-digit multiplication (Verguts and Fias, 2005). We show that, in many regimes, the semi-analytical and modified Runge Kutta methods outperform the Euler method, in some regimes by more than three orders of magnitude. Given the difference in execution time of the methods, and that the EM is widely used, we conclude that the researchers in the field can greatly benefit from our analysis.

## Methods

In the connectionist and dynamic field frameworks, the propagation of activation is a dynamic process operating in a continuous state space and evolving continuously over time (Grossberg, 1982; Thelen et al., 2001; Munakata and McClelland, 2003). In models that simulate this gradual activation process, it is typically formalized as a differential equation relating the rate of change of some variable (such as the activation of some unit) to the inputs it is currently receiving from other units via weighted connections. Equations describing the dynamic behavior of each unit are of the form

(1) $$\begin{array}{l} \dot{u}\left(t\right)=a\left(t\right)*u\left(t\right)+b\;\left(t\right)\equiv F\left(u,t\right) \end{array}$$

where *u*(*t*) describes the activation of one unit. *a*(t) and *b*(*t*) are weighted sums over all other units, governed each one by an equation of this form. *a*(*t*) also contains a spontaneous decay term, i.e., *a*(*t*) = −1 + … In Appendix A we discuss how connectionist and dynamic field equations can be reorganized to have the form of Equation (1) using two specific examples. We take advantage of the fact that both models have this form to develop optimum integration procedures that can potentially be applied to integrate both models, since the procedures we present here can in principle be applied to any set of coupled equations with the form given by Equation (1).

On the one hand, the Euler Method (EM) to solve Equation (1) is given by

(2) $$\begin{array}{l} u\left(t+dt\right)=u\left(t\right)+F\left(u,t\right)*\;dt \end{array}$$

where *dt* is the integration time-step.

On the other hand, to apply the fourth-order Runge-Kutta method (RK4M) to solve Equation (1) we need to calculate the coefficients *a*(*t*) and *b*(*t*) many times at each step. Since these coefficients are weighted sums over all other units, the bulk of the computing time is expended on these terms, at a great computational cost. The improved accuracy by the application of the RK4M comes with the cost of computing *a*(*t*) and *b*(*t*) to obtain k2, k3, and k4. To avoid this calculation, in the modified version we apply the original algorithm to calculate k1, but we only update the value for *u*(*t*) for the calculation of k2, k3, and k4. If, in Equation (1), we explicitly write the dependency of *F* on *a*(*t*) and *b*(*t*), the modified RK4M obeys

$$\begin{array}{l} k1\left(t\right)=F\left[t,u\left(t\right),a\left(t\right),b\left(t\right)\right]\\ k2\left(t\right)=F\left[t+\frac{dt}{2},u\left(t\right)+k1*\frac{dt}{2}\;,a\left(t\right),b\left(t\right)\right]\\ k3\left(t\right)=F\left(t+\frac{dt}{2},u\left(t\right)+k2*\frac{dt}{2},a\left(t\right),b\left(t\right)\right)\\ k4\left(t\right)=F\left[t+dt,u\left(t\right)+k3*dt,a\left(t\right),b\left(t\right)\right] \end{array}$$

and, as usual, *u*(*t* + *dt*) = *u*(*t*) + dt^*^(k1 + 2k2 + 2k3 + k4)/6.

Finally, for the derivation of the semi-analytical method (SAM), note that, in the particular case in which *a*(*t*) = *cte* = *a* and *b*(*t*) = *cte* = *b*, Equation (1) has analytical solution:

(3) $$\begin{array}{l} f\left(t+dt\right)=\left(f\left(t\right)+\;\frac{b\left(t\right)}{a\left(t\right)}\right)*\;e^{a\left(t\right)\;*dt}-\frac{b\left(t\right)}{a\left(t\right)} \end{array}$$

Even though *a*(*t*) and *b*(*t*) are not constants, the implementation of this “analytical” solution at each step of integration converges to the solution when *dt* goes to zero. One of us (AR) used a similar approach to study optical amplifiers' dynamic behavior (Rieznik and Fragnito, 2004), also governed by equations of the form of Equation (1). The analogy with an amplified signal can be more easily understood by rearranging Equation (3) as

(4) $$\begin{array}{l} f\left(t+dt\right)=f\left(t\right)\;*G\left(t\right)+\frac{b\left(t\right)}{a\left(t\right)}\left(G\left(t\right)-1\right) \end{array}$$

where *G*(*t*) = exp[*a*(*t*)^*^dt] is the signal gain. In Equation (4), the first term of the right-hand side represents the input activation multiplied by the gain or absorption that all other units produce on the activated unit. The second term is the noise term, named this way because it is present even in the absence of an input signal, and it is not modulated by this input. In fact, using this analogy, the effects of all other units on the activation of one given unit can be divided into two contributions: the noise contribution and the contribution that amplifies or absorbs the input signal.

We implement the SAM, the EM, and the modified RK4M to solve the IN model equations (Verguts and Fias, 2005), described in Appendix B. IN is a connectionist model of retrieval in single-digits multiplications, an area of interest for our research group (Zimmerman et al., 2016; Rieznik et al., 2017). The most important facts of arithmetic retrieval are (Verguts and Fias, 2005; Zimmerman et al., 2016): the problem size effect (small multiplication problems are easier than larger ones; cf. 3 × 2 and 7 × 8), the five effect (problems with five are easier than can be accounted for by their size), and the tie effect (problems with identical operands are easier than other problems; cf. 8 × 8 and 8 × 7). The basic assumption is that candidate answers to a particular problem are in cooperative/competitive interactions, and these interactions favor small, five, and tie problems.

## Results

To compare the three methods, we simulate the activation patterns of the network for the input 7 × 8, the most difficult operation in the multiplications table. The IN model describes a total of 107 coupled equations (see Appendix B): eight for each of the two-input fields (varying from 2 to 9), 36 semantic fields, nine tens fields (from 0 to 8), 10 units fields (from 0 to 9), and 36 response fields. To measure the accuracy of a simulation, we compute an average error over the response fields: if *u*_*n*_ is the numerical solution and *u*_*r*_ is the real one, the error is given by

(5) $$\begin{array}{l} error=sum\left(\left|u_{n}-u_{r}\right|\right)/36 \end{array}$$

Observe that the sum is performed over the 36 response fields, which explains why we divide by 36 to compute the error. Here, the real solution *u*_*r*_ is obtained using a very small *dt* (in our case, 10^−9^). We checked that the three methods converge to the same *u*_*r*_ solution.

**Figure 1:**
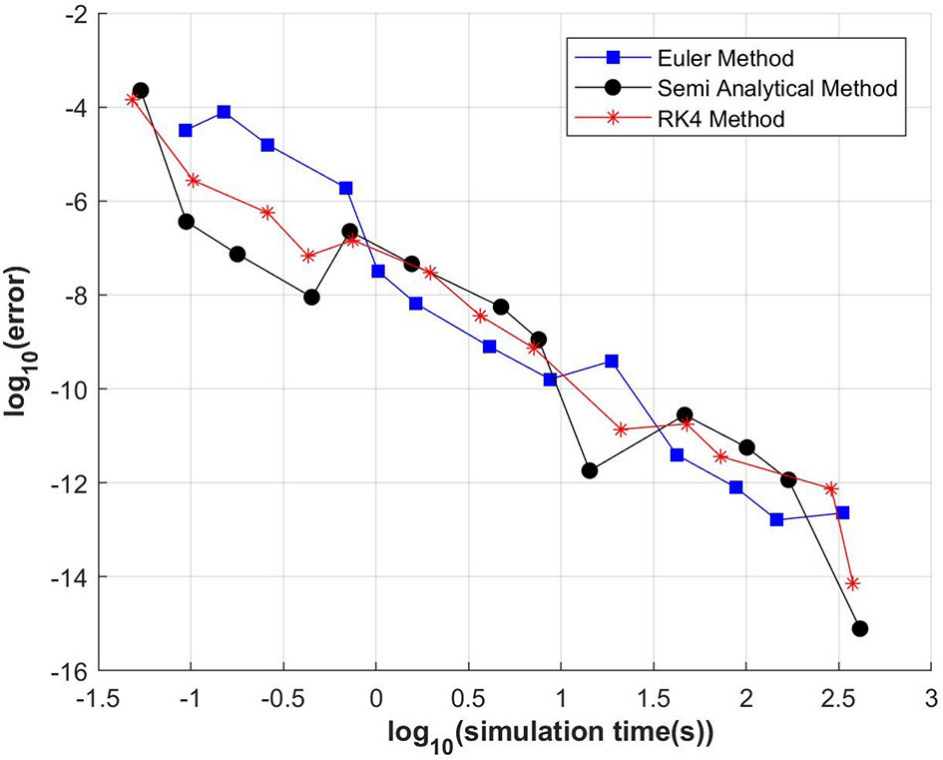
Simulation times vs. error for three methods: EM, SAM, and modified RK4M. Results varying *dt* from 10^−4^ to 10^−7^ are shown.

We use the following input parameters: *B* = 20, *M* = 10, *S* = 2, tao = 19.5, alpha = 0.75, and *C* = 0.5. It was shown in Verguts and Fias (2005) that these parameters fit human performance of retrieval in single-digits multiplications. All 107 input fields were zero at *t* = 0, except the first-operand input field for 7 and the second-operand input field for 8, which values were 20 (*B* = 20). After *t* = 0 it is assumed that the input stimuli are no longer present. The simulation is stopped at *t* = 0.1 (a.u.), which is the time that, for these parameters, it takes to the model to provide an answer (56, i.e., the result of 7 × 8). All simulation durations, given in seconds, were taken in a Yoga-Lenovo personal computer with an i7 intel processor running Matlab 2008 for windows. The Matlab codes are available at https://github.com/arieznik/IN_simulations (see Appendix C for instructions). Results are shown in Figure 1. In order to vary the simulation times and errors, we vary *dt* from 10^−4^ to 10^−7^. The exponent *j* defining the step size (*dt* = 10^−*j*^), was varied in steps of size 1 from 4 to 7 and for each of these values we also run two extra simulations, one with *dt* = 0.5^*^10^−j^ and the other with *dt* = 0.25^*^10^−j^.

It can be observed, in Figure 1, differences larger than three orders of magnitudes among the methods, but no method is the most efficient in all regimes. For any required error, the SAM or EM are faster than the RK4, in most cases by more than one or even two orders of magnitude. Equivalently, they are more precise given any required simulation time. Importantly, for commonly used errors, larger than ~10^−8^, the EM method is overperformed by the SAM methods in some cases by more than three orders of magnitude.

## Discussion

The fact that connectionist and DFT models can be written in the form of Equation (1) suggests that these results could be generalized to the simulations of other similar models, simpler or more complex. In the case here analyzed there are up to three orders of magnitude of difference in execution time among the models, but, despite that, the EM is the one that it is, to the best of our knowledge, universally used. Researchers in the field can benefit from these results: the two methods we introduce here can be easily implemented in open software like *cedar*; the matlab codes we share in our github repository (https://github.com/arieznik/IN_simulations) are well-commented and easy to follow in order to facilitate other researchers' further investigations on the regimes under which these methods overperform the EM in areas which are not the areas we are interested in (the interacting neighbors model for mathematical cognition).

These outstanding differences in simulation times using different methods also pose a question: how could a naïve user of the methods decide which one to use? Step-doubling is a well-known technique to estimate the local error and could be used to choose among methods (Gear, 1971).

In modeling optical amplifiers, adaptive step size selection criteria are used (Sinkin et al., 2003; Heidt, 2009; Rieznik et al., 2012). The integration step size (*dt* in this paper) varies along with the simulation according to a required-by-the-user local error. Step-doubling and linear extrapolation is used to obtain the higher-order solution (Gear, 1971). To the best of our knowledge, although widely used in other fields, these techniques have not been previously used, or even seriously investigated in simulations of the connectionist and DFT frameworks with applications in robotics. Although adaptive step size is not suitable when the evolution in time of sensory readings must be monitored, it may greatly improve simulations performances in other areas of robotics research. Under this framework, the user sets a target error and not a fixed step size.

In conclusion, we presented preliminary results suggesting that the two alternative methods to the EM for the integration of connectionist and dynamic field models (the SAM and modified RK4M that we developed here) may greatly improve the simulations, but further investigation is necessary to understand the regimes under which the application of the SAM or RK4M is preferable over the EM, which is, to the best of our knowledge, the default method used by researchers.

## Data Availability Statement

The raw data supporting the conclusions of this article will be made available by the authors, without undue reservation.

## Author Contributions

AR: project supervision and methodology. AR, RD, LS, and AB: materials development, software development, and validation. AR and AB: data reduction, data analysis, and writing. RD: writing. All authors contributed to the article and approved the submitted version.

## Conflict of Interest

The authors declare that the research was conducted in the absence of any commercial or financial relationships that could be construed as a potential conflict of interest.

## Appendix

**Appendix A: Reorganizing connectionist and dynamic field theory equations as Equation (1)**

We show next two examples on how to reorganize model equations in order to obtain the form of Equation (1). The two examples are one connectionist model (Verguts and Fias, 2005) and one dynamic field model (Thelen et al., 2001). We begin by the later.

Equation (8) in Thelen et al. (2001) is

(A1) $$\begin{array}{l} \tau \dot{u}\left(x,t\right)=\;-u\left(x,t\right)+S\left(x,t\right)+\int w\left(x-x^{\prime }\right)f\left(u\left(x^{\prime }\right)\right)dx^{\prime }\\ \;+h+q\xi \left(x,t\right) \end{array}$$

If we define *a*(*x, t*) = −1/ τ and

(A2) $$\begin{array}{l} \tau b\left(x,t\right)=\;S\left(x,t\right)+\;\int w\left(x-x^{\prime }\right)f\left(u\left(x^{\prime }\right)\right)dx^{\prime }+h+q\xi \left(x,t\right) \end{array}$$

and we drop the *x* dependence, (A1) is

(A3) $$\begin{array}{l} \dot{u}\left(t\right)=\;a*u\left(t\right)+\;b\left(t\right) \end{array}$$

which has the form of Equation (1), as we want to demonstrate.

Note that the Equation (A3) is linear because not *a**u(*t*) nor *b*(*t*) contain non-linear terms [*f* (*u*) in Equation (A2) is a step-function which equals 1 or 0 depending on the *u* value]. The example we give next, however, the connectionist IN model equations, is non-linear, since, as we shall see, the term *a*(*t*)**u*(*t*) contain non-linearities. The IN model equations are explained in Appendix B. For the semantic fields, Equation (B2), we have

(A4) $$\begin{array}{l} \frac{d}{dt}y_{i}\left(t\right)=-y_{i}\left(t\right)+\underset{j}{\sum }w_{ji}^{in}x_{j}\left(B-y_{i}\left(t\right)\right) \end{array}$$

which can be reorganized as

(A5) $$\begin{array}{l} \frac{d}{dt}y_{i}\left(t\right)=-y_{i}\left(t\right)\left(1\;+\underset{j}{\sum }w_{ji}^{in}x_{j}\right)+\sum_{j}w_{ji}^{in}x_{j}B \end{array}$$

Now, defining

$$\begin{array}{l} a_{i}\left(t\right)=-\left(1\;+\underset{j}{\sum }w_{ji}^{in}x_{j}\right) \end{array}$$

and

$$\begin{array}{l} b_{i}\left(t\right)=\sum_{j}w_{ji}^{in}x_{j}B, \end{array}$$

And dropping the subscript *i*, Equation (A4) can be rewritten as

$$\begin{array}{l} ẏ\left(t\right)=\;a\left(t\right)*y\left(t\right)+\;b\left(t\right) \end{array}$$

Which, as we want to demonstrate, has the form of Equation (1). Observe that *a*(*t*)*y(*t*) contains non-linearities, since *a*(*t*) is linear on the other units and then *a*(*t*)*y(*t*) contains the multiplication of two units' values. In Table A1 we show the expressions for *a*(*t*) and *b*(*t*) for the other fields in the IN model.

**Table A1:** Coefficients *a*(*t*) and *b*(*t*) for each field in the IN model equations.

**(Equation) Field**	**a_***i***_(t)**	**b_***i***_(t)**
(B1) input field	−(1 +*I*_*i*_(*t*))	*BI*_*i*_(*t*)
(B2) semantic field	$-\left(1\;+\sum_{j}w_{ji}^{in}x_{j}\left(t\right)\right)$	$B\underset{j}{\sum }w_{ji}^{in}x_{j}\left(t\right)$
(B3) decade field	$-\left(1\;+M\underset{j}{\sum }w_{ji}^{T}y_{j}\left(t\right)+\underset{j\neq i}{\sum }z_{j}^{T}\left(t\right)\right)$	$BM\underset{j}{\sum }w_{ji}^{T}y_{j}\left(t\right)$
(B4) unit field	$-\left(1\;+M\underset{j}{\sum }w_{ji}^{U}y_{j}\left(t\right)+\underset{j\neq i}{\sum }z_{j}^{U}\left(t\right)\right)$	$BM\underset{j}{\sum }w_{ji}^{U}y_{j}\left(t\right)$
(B5) response field	$-\left(1\;+\underset{j}{\sum }w_{ji}^{R}z_{j}\left(t\right)\right)$	$B\underset{j}{\sum }w_{ji}^{R}z_{j}\left(t\right)$

**Appendix B: IN model equations**

Note: the equation here shown had some typos in the original article in which they were presented (Verguts and Fias, 2005). The authors sent us, through a personal communication, a corrected version, which we explain next.

For each of the two input fields, for an input unit i at time t, activation xi of that unit at time t is described by

(B1) $$\begin{array}{l} \frac{d}{dt}x_{i}\left(t\right)=-x_{i}\left(t\right)+I_{i}\left(t\right)\left(B-x_{i}\left(t\right)\right) \end{array}$$

In Equation (A1), –*x*_i_(*t*) functions as a decay term. The factor *I*_i_(*t*) is an indicator function which, at time *t*, equals one if number *i* was presented in its input field and zero otherwise. The parameter B is an upper bound to the activation values *x*_i_. Hence, the combination of the decay term and the *I*_i_(*t*)[B – *x*_i_(*t*)] terms restrict *x*_i_ between zero and B.

The activation *y*_i_ of a semantic unit *i* has the following equation:

(B2) $$\begin{array}{l} \frac{d}{dt}y_{i}\left(t\right)=-y_{i}\left(t\right)+\sum_{j}w_{ji}^{in}x_{j}\left(B-y_{i}\left(t\right)\right) \end{array}$$

In Equation (B2), the sum is taken over all nodes in both input fields. Again, the factor B – *y*_i_ bounds the equation below B. The equation is also bounded above zero: For example, in the absence of any input, the equation becomes d*y*_i_(*t*)/dt = –*y*_i_(*t*), and *y*_i_ decays exponentially toward zero. The connection weight *w*^*in*^_*ji*_ are given by *w*^*in*^_*ji*_ = exp[–α * abs(*i*–*j*)] – C, where α and C are two input parameters for the model.

The equation describing activation *z*_*i*_^T^ of unit *i* in the decade field (with superscript T for tens, or decades) is

(B3) $$\begin{array}{l} \frac{\text{d}}{\text{dt}}z_{i}^{T}\left(t\right)=-z_{i}^{T}+M\sum_{j}w_{ji}^{T}y_{j}\left(B-z_{i}^{T}\left(t\right)\right)-z_{i}^{T}\sum_{j\neq i}z_{j}^{T} \end{array}$$

and similarly, for the units field, with superscript U for units,

(B4) $$\begin{array}{l} \frac{\text{d}}{\text{dt}}z_{i}^{U}\left(t\right)=-z_{i}^{U}+M\sum_{j}w_{ji}^{U}y_{j}\left(B-z_{i}^{U}\left(t\right)\right)-z_{i}^{U}\sum_{j\neq i}z_{j}^{U} \end{array}$$

The weights *w*_*ji*_^*T*^ and *w*_*ji*_^*U*^ are zero or one depending on whether node *j* should feed into decomposition field unit *i* or not. For example, the weight from the 24-node in the semantic field to the twenties node in the decades field is one, to the thirties node it is zero, and so on.

In the response field, for node *i* with activation *r*_i_(*t*),

(A5) $$\begin{array}{l} \frac{\text{d}}{\text{dt}}r_{i}\left(t\right)=-r_{i}\left(t\right)+\sum_{j}w_{ji}^{R}z_{j}\left(B-r_{i}\left(t\right)\right) \end{array}$$

The weights *w*_*ji*_^*R*^ are zero or S, again depending on whether the connection between the decomposition (decade or unit) node and the response node should be formed or not. The parameter S is a scaling parameter which influences the overall speed of responding in the network.

In addition to these parameters, there is also a threshold that has to be reached before a response is executed, which is denoted alpha. To sum up, the parameters used in the model are B, M, S, and alpha, in addition to the parameters α and C describing the connection weight between two semantic fields. The first four of these are scaling parameters, in the sense that changing their values changes the simulated RTs (e.g., RTs are generally slower with a higher threshold alpha), but does not change the qualitative pattern of predictions.

**Appendix C: Download, installation and running the codes**

You must save the compressed folder called INmodel.zip in your computer. When you extract it, five matlab files (.m) will be created inside the folder IN_model:

- IN_SAM.m, IN_EM.m, and IN_RK4.m: these functions use the SAM, EM, and RK4 methods to solve the IN equations using as inputs the first operand (op1), the second operand (op2 ≤ op1), alpha, C, and dt. Be sure of adding these functions to the matlab path before the simulation starts.
- IN_Simulation.m: this is the file you must run in order to perform the simulations. By default, it runs the simulation using op1 = 8 and op2 = 7, since in this article we simulated the pattern of activations for the input 8 × 7. It also uses by default the IN_SAM.m function. It is easy to change the problem to be simulated (for instance, to 8 × 8) or the method used for the integration since the code is well-commented.
- Figure_1.m: if you run this file, after a few seconds Figure 1 in this article will pop put.

Apart from these .m files, a set of .mat files will be created inside the IN_model folder. These are matlab variables containing the results of the simulations using the three methods here presented for the input 8 × 7 and with dt varying from 10-4 to 10-7. These files are used when ran Figure_1.m in order to generate Figure 1. Be sure they are included in the matlab path when running this file.
